# Mortality trends for accidental falls in older people in Spain, 2000-2015

**DOI:** 10.1186/s12877-017-0670-6

**Published:** 2017-11-28

**Authors:** Alicia Padrón-Monedero, Javier Damián, M. Pilar Martin, Rafael Fernández-Cuenca

**Affiliations:** 1 National Centre for Epidemiology. “Carlos III” Institute of Health, Av Monforte de Lemos 5, 28029 Madrid, Spain; 2 CIBERNED (Consortium for Biomedical Research in Neurodegenerative Diseases, Madrid, Spain; 3 Adelphi University, College of Nursing and PH, Garden City, NY 11530 USA; 4 CIBERESP (Consortium for Biomedical Research in Epidemiology and Public Health, Madrid, Spain

**Keywords:** Aged, Falls, Mortality, Time series

## Abstract

**Background:**

Accidental falls in older people are a major public health problem but a relatively limited number of studies have analyzed the mortality trends from this cause. Effective public health interventions have been found to prevent the incidence of falls and their complications. Therefore, characterizing the mortality trends of falls for different subpopulations can help to identify their needs and contribute to develop more appropriate prevention programs for specific target groups.

**Methods:**

This study was based on a longitudinal analysis of death rates from accidental falls (2000-2015) stratified by sex for the population ≥ 65 years and by age groups (65-74, 75-84, ≥85). A joinpoint regression model was used to identify trend inflection points. The Annual Percent Change (APC) was estimated for each trend.

**Results:**

Mortality rates per 100,000 person-years increased from 20.6 to 30.1 for men and 13.8 to 20.8 for women between 2000 and 2015. Men presented a relevant trend increase between 2008 and 2015 (APC [95% CI] 7.2% [5.3;9.2]) and women between 2008 and 2013 (7.9% [4.1;11.8]) There were no trend differences between sexes. For 65-74 years old men we found a relevant increase in the last period (2011-2015) (7.8% [1.0;15.1]). Those aged 75-84 years showed a trend increase between 2007 and 2015 (6.4% [4.4;8.4]) and men ≥85 years presented a remarkably high trend between 2008 and 2015 (9.0% [5.2;13]). There were no relevant differences between age groups. Women aged 65-74 had no relevant trend through the period. Those aged 75-84 presented an uniform trend increase for the whole period, 2000-2015, (3.4% [2.3;4.4]) and women ≥85 had and important trend increase between 2008 and 2013 (11.1% [5.3;17.2]), that has reached an stable level in the last 2 years. There were no relevant differences between the 75-84 and ≥85 age groups.

**Conclusions:**

Recent mortality trends from accidental falls increased in men ≥65 years and women ≥75 years. These results recommend the implementation of specific preventive programs.

## Background

Accidental falls in older people are a major public health problem and a priority for intervention for WHO/Europe [1]. Approximately one third of individuals 65 years and older fall each year [2] Accidental ground level falls have significant consequences on mortality and morbidity in older people [3, 4].

In the European Union, people over 65 account for half of the deaths from unintentional injuries despite representing only 20% of the population [5]. Unintentional injuries represent between the fifth and the seventh leading cause of death in older adults in developed countries [6, 7] and most of these injuries are caused by falls [2, 7, 8].

Despite the importance and magnitude of falls in older people in developed countries, a relatively limited number of studies have analyzed the mortality trends from this cause [8–16] and most of them have reported an increase. Furthermore, the progressive aging of the population makes it likely that the magnitude of mortality from falls will increase in the future [3, 9, 17]. However, up to date, no studies have characterized these trends in Spain, and few have assessed them recently in Europe [15].

Effective public health interventions have been found to prevent the incidence of falls and their complications [18, 19]. Consequently, characterizing the mortality trends of falls for different subpopulations can help to identify the needs of these groups and contribute to develop appropriate prevention programs for specific target groups.

The aim of this study is to measure the mortality rates from accidental falls and analyze its trends over the last 15 years (2000-2015) among adults 65 years and older and the different subpopulations in Spain.

## Methods

### Data source

The information source has been the microdata from the Death Statistics by cause of death provided by the Spanish National Statistics Institute. In Spain when the death is due to an external cause, the information is recorded in the Boletín Estadístico de Defunción Judicial (Judicial Statistical Death Bulletin) for data collected after 2009, and in a different document (MNP-52) for data prior to that year. To ensure confidentiality of the patients, we used anonymized data. The inclusion criteria were national deaths from Spain’s residents over 64 years whose cause of death was an accidental fall between 2000 and 2015. The codes of the International Classification of Diseases (ICD) for an accidental fall were the W00 to W19 of the ICD-10.

### Statistical analyses

Age adjusted mortality rates for the study population (≥ 65 years), for each sex, were calculated by using the direct method and the standard European population recommended by Eurostat as a reference [20]. Crude mortality rates per 100,000 person-years were computed for the different age groups (65-74, 75-84 and ≥85) using population data provided by the Spanish National Statistics Institute.

The trends of death rates were analyzed by using generalized linear models and assuming a Poisson distribution [21]. The analysis initially assumes a joinpoint regression model of zero joinpoints and iterative fits alternative models, by using permutation test to identify the optimal curve with the fewer number of joinpoints, setting a maximum limit of three. This method allows to identify trend inflection points (years) and the annual percent change (APC) with its confidence intervals for each of the trends [21]. The APC was calculated as APC = 100 × (exp (*β*) - 1) where *β* is the slope of the regression line.

In order to analyze and compare trend differences among population subgroups we estimated the Average Annual Percent Change (AAPC) of the overall period for the different subgroups of sex and age by using joinpoint regression models. The AAPC takes into account the APC of segmented analysis (APCs) to summarize and compare the trend of a given period taking into consideration the possible trend inflection points of the sub-periods [22]. The AAPC was calculated as AAPC = 100 × (exp (∑ *w*_*j*_ × *β*_*j*_) − 1), where *β*_*j*_ are the slopes of each trend and *w*_*j*_ are weights proportional to the length of the time partitions. The APC, is not the optimum measurement to compare trends for different subpopulations, because depends on the length of the interval. When a summary measurement of a trend is needed over a specified time interval, the AAPC provides an essential complement to the more detailed results provided by the APC, and is considered a more adequate measurement to compare trends between different subpopulations [22].

The data were analyzed using Stata 14 (StataCorp LP, College Station, Texas 77,845 USA) and Joinpoint Regression Version 4.2.0 (April 2015; Statistical Methodology and Applications Branch, Surveillance Research Program, National Cancer Institute) programs.

## Results

In Spain, between 2000 and 2015, there were 30,893 accidental fall deaths, with an annual average of 1042 deaths in men and 889 in women. Of those, 23,502 (10,506 men and 12,996 women) occurred in persons aged ≥65, representing the 76.1% of total accidental fall fatalities for all ages (63.0% in men and 91.4% in women). Total mortality rates per 100,000 person-years for accidental falls were 5.2 for men and 3.4 for women. However, rates for those ≥65 years were 20.3 and 18.6 respectively.

**Fig. 1:**
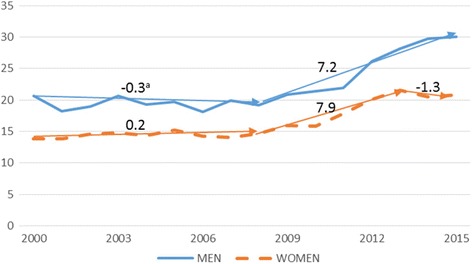
Adjusted mortality rates (per 100,000 person-years) by accidental falls in older people by sex. 2000-2015. ^a^Annual Percentage Change (APC) in older people by sex, corresponding to Table 1 (men) and Table 2 (women)

Adjusted mortality rates in older adults have risen from 16.3 per 100,000 person-years in 2000 to 24.6 in 2015. In men, adjusted rates rose from 20.6 to 30.1 per 100,000 person-years and in women they rose from 13.8 in 2000 to 20.8 in 2015. AAPC increases in mortality trends were relevant for both sexes, with AAPC differences between men and women highly compatible with no difference (AAPC difference 0.6% (95% CI:-1.5; 2.7)). This trend similarity for both sexes is shown in Fig. 1.

The analyses in men aged ≥65 identified one trend inflection point during the whole 2000-2015 period, that virtually corresponds with the first trend inflection point found in women (Tables 1 and 2). An important increase in mortality trend was identified between 2008 and 2015 with APC of 7.2%.

**Table 1:** Mortality trends for men. Trend inflection points, APC for each period and AAPC (overall period)

	Overall period 2000-2015		1st trend			2nd trend		
Age group	AAPC (95% CI)	*p*	Period	APC (95% CI)	*p*	Period	APC (95% CI)	*p*
All (≥65)^a^	3.1 (1.9;4.3)	<0.001	2000-2008	−0.3 (−2.3;1.6)	0.700	2008-2015	7.2 (5.3;9.2)	<0.001
65-74^b^	2.2 (0.3;4.1)	<0.001	2000-2011	0.2 (−1.4;1.9)	0.800	2011-2015	7.8 (1.0;15.1)	<0.001
75-84^b^	3.5 (1.9;5.0)	<0.001	2000-2007	0.2 (−2.7;3.2)	0.900	2007-2015	6.4 (4.4;8.4)	<0.001
≥85^b^	3.3 (0.7;5.8)	<0.001	2000-2008	−1.5 (−5.5;2.6)	0.400	2008-2015	9.0 (5.2;13)	<0.001

*AAPC* Average Annual Percent Change, *APC* Annual Percent Change, *CI* Confidence Interval

^a^For the total population, trends have been assessed with age-adjusted rates and standard errors

^b^For each age group trends have been assessed with crude mortality rates by taking the number of deaths and population data assuming a Poisson model distribution

**Table 2:** Mortality trends for women. Trend inflection points, APC for each period and AAPC (overall period)

	Overall period 2000-2015		1st trend			2nd trend			3rd trend		
Age group	AAPC (95% CI)	*p*	Period	APC (95% CI)	*p*	Period	APC (95% CI)	*p*	Period	APC (95% CI)	*p*
All (≥65)^a^	2.5 (0.8;4.2)	<0.001	2000-2008	0.2 (−1.3;1.7)	0.800	2008-2013	7.9 (4.1;11.8)	<0.001	2013-2015	−1.3 (−10.5;8.8)	0.800
65-74^b^	1.1 (−0.1;2.2)	0.100	2000-2015	1.1 (−0.1;2.2)	0.100						
75-84^b^	3.4 (2.3;4.4)	<0.001	2000-2015	3.4 (2.3;4.4)	<0.001						
≥85^b^	2.5 (0.0;5.1)	<0.001	2000-2008	−1.1 (−3.5;1.3)	0.300	2008-2013	11.1 (5.3;17.2)	<0.001	2013-2015	−2.9 (−15.6;11.7)	0.600

*AAPC* Average Annual Percent Change, *APC* Annual Percent Change, *CI* Confidence Interval

^a^For the total population, trends have been assessed with age-adjusted rates and standard errors

^b^For each age group trends have been assessed with crude mortality rates by taking the number of deaths and population data assuming a Poisson model distribution

Table 1 shows the joinpoint analysis for men in the three age groups. For those between 65 and 74 years, there was one inflection point with an increase in APC for the last period (2011-2015) of 7.8%. For those between 75 and 84 years, one inflection point showed a clear trend increase between 2007 and 2015 with an APC of 6.4%. Likewise in those older than 84 years, one trend inflection point was identified with an APC of 9.0%, for the period 2008-2015.

To perform comparisons between groups we computed AAPCs. The differences in mortality trends between the age groups were negligible (differences in AAPC between the 75-84 and 65-74 years old groups of 1.3% (95% CI: -1.2;3.8), between the ≥85 and 65-74 years old groups of 1.1% (95% CI: -2.1;4.3) and between the 75-84 and ≥85 years old groups of −0.2% (95% CI: -3.2;2.8)).

Table 2 shows results for women. In those aged ≥65, two trend inflection points were identified, resulting in one large increase, with an APC of 7.9% for the period 2008-2013. For those women 65-74 years old, no inflection points were identified with a non-relevant APC for the whole period. Likewise, in women 75-84 years old, no inflection points were identified, but they had a relevant APC for the whole period 2000-2015 of 3.4%. And for those 85 years and older, two trend inflection points provided a relevant trend between 2008 and 2013 with ​​APCs of 11.1% followed by a stable level in the last 2 years (2013-2015) with an APC of −2.9%, yet with a wide confidence interval.

For women, the differences in mortality trends between the age groups were not relevant (difference in AAPC between 75 and 84 and ≥85 years old groups of −0.8% (95% CI: -3.5; 1.9)).

**Fig. 2:**
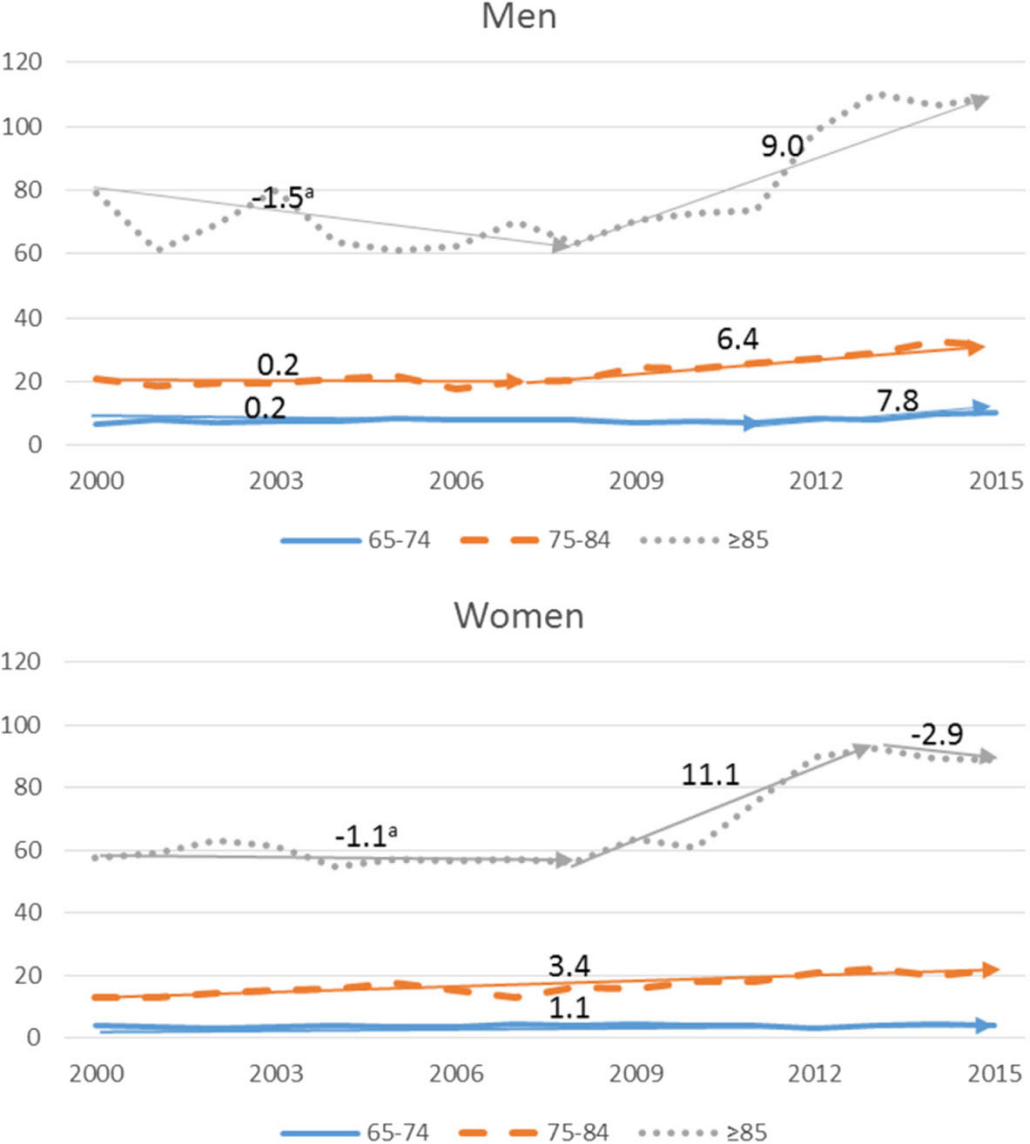
Crude mortality rates in older people by accidental falls by age group and sex. 2000-2015. ^a^Annual Percentage Change (APC) in older people, corresponding to Table 1 (men) and Table 2 (women) by age group and sex

Figure 2 shows the graphical representation of the evolution of mortality rates from accidental falls for the different age groups, stratified by sex, where it can be noticed an increase in the rates when increasing the age group. In those 75-84 and ≥85 years old, the differences in AAPC were not relevant between the sexes with AAPCs of 0.1% (95% CI:-1.7;1.9) and 0.7% (95% CI: -2.9;4.3) respectively.

## Discussion

National mortality trends (2000-2015) for accidental falls in people aged 65 years or older show an increase for the overall population, for men of all age groups and for women ≥75 years. The last period of change in mortality trends shows a relevant increase and the APCs are the largest of the last 15 years for men of all ages and women aged 75 years or older, with a mild stabilization in the last 3 years for women ≥85 years.

Our results are consistent with the increases in mortality rates from accidental falls found in other studies for people aged ≥65, [9–11, 13, 14, 23] in both sexes [9, 13, 14, 23]. Interestingly, for the total population ≥ 65 years, we did not identify sex differences in mortality trend increases, although the mortality rates for all years and age groups were higher for men, which is consistent with other studies [2, 9]. However, data from both Spain and other developed countries show that the accident rate for those 65 and older is higher for women [24], which apparently does not translate into a greater lethality [2, 14, 19]. The higher mortality rate identified for men might be partly explained because men suffer from more comorbidities and poorer health than women of the same age, which could worsen the harmful consequences of fractures [14, 19]. However, although Hindmarsh et al. have suggested that a greater number of comorbidities are associated with a higher lethality due to fractures caused by falls in older people, the excess mortality from hip fractures in men persists even after adjusting for comorbidities [25, 26]. This excess of mortality rates from accidental falls in men over 64 years compared with women deserves further study.

It has been described that mortality due to accidental falls in older people increases with age [3, 9, 13, 17]. Other studies have shown that mortality trends due to accidental falls among older people increases in almost all age groups [10] but as far as we know, no study has made ​​a comparative analysis of mortality trends among the different age groups using the AAPC provided by the joinpoint regression models. Our results, stratified by age group, show that in recent years, the increase in mortality trends appears to be attributable mainly to the group of 75 years and older in women and to all groups ≥65 in men. For women 65 to 74 years old mortality rates tend to a modest non-relevant increase. Thus, it is likely that the differences in mortality trends caused by accidental falls increase in the future between subpopulations of women 65-74 years old and 75 and older. Additionally, in recent years, women 75 to 84 years old have shown a relevant increase in mortality trends, although of lower magnitude than for the older age group. In the ≥85 year old group, after a remarkable increase of mortality trends, the stable level reached in the last 2 years (2013-2015) deserves further follow up.

Possible causes of the increases in mortality trends for accidental falls in the overall population aged 65 years or older could be: 1) An increase in the incidence of falls, 2) a better recording of deaths by this cause, 3) a more severe type of falls 4) an increased prevalence of frailty. We have no evidence to consider that have been changes in healthcare in the country that could cause this increase.

Concerning the first hypothesis, we have assessed that the incidence of accidents in the last 10 years, did not only fail to increase for this population, but has fallen, according to the Spanish National Health Survey [27]. Moreover, the exploratory analysis of hospital admissions rates in Spain, in older adults with a diagnosis of an accidental fall (ICD-9-CM E880-E888) in the last 5 years, have shown decreases in the case of women (APCs of −0.6%, −0.6%, and −0.8% for those 65-74, 75-84 and ≥85 years old respectively), and APCs of −1.1%, 0.5% and 1.3% for men 65-74, 75-84 and ≥85 years old respectively (data not shown). It is remarkable that the hospital admissions for accidental falls for men between 65 and 74 years old, have decreased −1.1% per year. Thus, the increase in mortality trends caused by falls of a much larger magnitude identified in recent years, for almost all subpopulations analyzed, it is unlikely that could be substantially attributed to an increase in the incidence of falls. These data are consistent with other studies that have found an increased mortality caused by accidental falls in the older population but not an increase in hospital admissions for fractures or falls in the same period [14]. Other studies have also shown an increase in death rates from accidental falls, but the rates of nonlethal falls did not increase significantly [10, 23] and the hospital admissions for this cause [10] have declined in the population aged 65 years and older, which is consistent with our results. As far as we know, only one study has shown an increase in hospitalizations due to falls in the elderly population of United States [28].

Regarding the second hypothesis, the data that we present correspond to a period in which the ICD-10 classification was uniformly stablished in Spain. Besides, we have not found a relevant increase in mortality trends for the subpopulation of women aged 65 to 74 years. These data apparently does not support the hypothesis that these increases in mortality trends are substantially due to a better recording of falls as the underlying cause of death in older people.

With regard to the hypothesis that increases in mortality trends could be due to changes in the severity of falls, our results show a recent increase in the mortality trends for men even in the younger age group (65-74 years). According to Sung et al. people aged 65-74 may have a better health and functional condition than previous cohorts making it easier to engage in activities that increase their risk of accidental falls [12] mainly in the case of men [14]. Furthermore, those aged less than 75 years tend to be more active, doing more activities and are more likely to suffer falls outside the home compared with older age groups, [14, 29] and these falls are supposed to be caused by more severe mechanisms. On the other hand, it has also been reported that minor falls in older people caused by a seemingly innocuous mechanism could produce disproportionately severe injuries, even death, compared to younger population [4]. According to Bath et al. falls within the home were associated with an increased mortality, to be 75 and older, to have less mobility and with indicators of greater frailty [29]. Perhaps for different age groups the severity of the consequences of the falls may have increased by different mechanisms. However, data from the last Spanish National Health Survey (2012) show that incidence of falls has declined over the last 10 years [27], but we have appreciated a fall mortality increase between 2011 and 2015 so the lethal consequences may have increased, perhaps due to different causes for each age group. It is advisable to assess if future trends maintain this mortality increase due to accidental falls with special focus in younger groups.

The hypothesis that the increased mortality from falls could be due to an increased frailty of these populations is consistent with a work by Baker et al. that suggests that the elderly population is at increased risk of mortality for minor injuries unlikely to cause death in younger populations [30]. According to Stevens et al. frailty in the elderly population could have increased in recent years, which is consistent with our results [16], mainly for men. This could be due to an improved survival for populations with poorer baseline health, a higher number of comorbidities and/or polymedicated for this cause, that after some external damage could trigger an imbalance of their condition of frailty making them more susceptible to falling mortality [16], which is consistent with previous studies [23, 25, 31]. Apparently, the improved survival in a population with a high number of comorbidities could place them in a condition of frailty [16]. On the other hand, it has been suggested that the association between age and mortality after hip fracture remains after adjusting for numerous comorbidities, therefore could be of interest to assess the impact on mortality of specific indicators of frailty (performance of daily activities, healthy attitudes, and variables related to function and nutrition), taking into account pertinent comorbidities [26]. Moreover, we cannot rule out that the economic crises that started in 2007 could have affected the frailty of the older population through different ways. Furthermore, we have assessed the stabilization in falls mortality in women ≥85 years old in the last 3 years. It is advisable to assess the development of future trends in older women.

The recent increase in the fall death rates in almost all the studied population subgroups is worrisome. In addition, the progressive aging of the population makes foreseeable that these figures will increase in the future. The differences in mortality trends among different demographic subpopulations suggest that a plausible explanatory hypothesis for this increment could be the increased frailty and/or the severity of falls in men ≥65 years and women 75 years and older; future studies would be needed to confirm the association between falls mortality in older persons with different indicators of increased frailty. Consequently, it would be advisable to implement public health programs both in the community and in the health care settings that prioritize the prevention and/or attenuation of the frailty mainly in the group of 75 and over. These could include, among others, an optimal control of comorbidities, medications, a healthy eating pattern [32] and the practice of moderate physical activity [19]. The prioritization of these activities should not exclude other effective additional interventions for fall prevention [19] in the group of 75 and more years and also in younger age groups, mainly in the case of men. Moreover, activities that reduce frailty could apparently reduce the incidence of falls in the older population [19].

### Strengths

This is a national study that has analyzed all deaths due to accidental falls in people 65 years and older between 2000 and 2015. By using joinpoint regression models, we have analyzed the trend inflection points in that period for the total population and the different subpopulations. This is the first study in Spain that has additionally compared these mortality trends for the different demographic subpopulations to analyze differences that could provide guidance on explanatory hypotheses.

### Limitations

First, it has not been possible to analyze the different causes of accidental falls due to codification issues. The most common code of death by an accidental fall for people 85 years old and older was “falls due to unspecified causes” (W19). This finding is consistent with previous studies [11]. The remaining death codes due to accidental falls were far less frequent. Therefore, all causes have been combined into a single group classified as accidental falls. Second, due to the frailty of older persons, even light accidental falls, could produce an imbalance leading to a deferred death ascribed to another cause [16].

## Conclusions

In recent years mortality trends from accidental falls have shown a relevant increase in men aged 65 years and older and in women 75 years and older. Our results suggest that implementing specific preventive programs could be beneficial to reduce the burden of accidental falls.

## Abbreviations

AAPC: Average Annual Percent Change
APC: Annual Percent change
ICD: International Classification of Diseases
